# Infill Density Influence on Mechanical and Thermal Properties of Short Carbon Fiber-Reinforced Polyamide Composites Manufactured by FFF Process

**DOI:** 10.3390/ma15103706

**Published:** 2022-05-22

**Authors:** Lucia-Antoneta Chicos, Mihai Alin Pop, Sebastian-Marian Zaharia, Camil Lancea, George Razvan Buican, Ionut Stelian Pascariu, Valentin-Marian Stamate

**Affiliations:** 1 Department of Manufacturing Engineering, Transilvania University of Brasov, 500036 Brasov, Romania; zaharia_sebastian@unitbv.ro (S.-M.Z.); camil@unitbv.ro (C.L.); buican.george@unitbv.ro (G.R.B.); ionut.pascariu@student.unitbv.ro (I.S.P.); valentin_s@unitbv.ro (V.-M.S.); 2 Department of Materials Science, Transilvania University of Brasov, 500036 Brasov, Romania; mihai.pop@unitbv.ro

**Keywords:** fused filament fabrication, carbon fiber, polyamide, infill density, mechanical properties, thermal properties

## Abstract

In three-dimensional (3D) printing, one of the main parameters influencing the properties of 3D-printed materials is the infill density (ID). This paper presents the influence of ID on the microstructure, mechanical, and thermal properties of carbon fiber-reinforced composites, commercially available, manufactured by the Fused Filament Fabrication (FFF) process. The samples were manufactured using FFF by varying the infill density (25%, 50%, 75%, and 100%) and were subjected to tensile tests, three-point bending, and thermal analyses by Differential Scanning Calorimetry (DSC) and Thermogravimetric Analysis (TGA). It was shown that the samples with 100% ID had the highest values of both tensile, 90.8 MPa, and flexural strengths, 114 MPa, while those with 25% ID had the lowest values of 56.4 MPa and 62.2 MPa, respectively. For samples with infill densities of 25% and 50%, the differences between the maximum tensile and flexural strengths were small; therefore, if the operating conditions of the components allow, a 25% infill density could be used instead of 50%. After DSC analysis, it was found that the variation in the ID percentage determined the change in the glass transition temperature from 49.6 °C, for the samples with 25% ID, to 32.9 °C, for those with 100% ID. TGA results showed that the samples with IDs of 75% and 100% recorded lower temperatures of onset degradation (approximately 344.75 °C) than those with infill densities of 25% and 50% (348.5 °C, and 349.6 °C, respectively).

## 1. Introduction

Additive Manufacturing (AM), in recent years, has become one of the emerging and sustainable manufacturing processes. Its ability to manufacture complex, lightweight parts with a short manufacturing time and low material consumption has led to its use in various industries such as automotive, medical, and aerospace, in the manufacture of large portions or components of aircraft, rockets, and unmanned aerial vehicles (UAVs). The Fused Deposition Modeling (FDM) process, known as Fused Filament Fabrication (FFF), which uses the hot melt and adhesive properties of thermoplastics, is the most applied 3D printing technology due to its flexibility, simplicity, and low cost in the production of complex shape parts used in various fields [1].

Thermoplastic polymers are materials frequently used in various industries, also in the FFF process related to additive manufacturing technology, due to their high resistance to cooling and heating cycles, resistance to chemicals and mechanical stress, ease of production, and low costs [2]. In order to improve the properties and characteristics of polymers manufactured by AM, filled polymers have begun to be developed in recent years, and the most used fillers are carbon fibers, glass, carbon nanotubes, nanoparticles, and synthetic and natural fibers [1,2,3,4,5]. Thermoplastic polymeric matrices commonly used in FFF are polylactides (PLA), acrylonitrile butadiene styrene (ABS), and polyamides (PA), such as PA6, PA12, and polyether-ether-ketone (PEEK). Although the additive manufacturing of polymer composites is considered to be cost-effective, it is still in its infancy because there are a number of problems that occur in the manufacturing process or factors that influence the process and therefore have an influence on the properties and characteristics of components manufactured by FFF.

The 3D printing of fiber-reinforced polymer composites has become of real interest and current research claims the need to develop the process and composite materials used in this process. The mechanical and thermal performance of composite fibers manufactured by FFF are influenced by a number of printing variables, matrix characteristics, fiber content, fiber orientation, fiber-matrix bonding, etc. In order to obtain components from high-strength fiber composites through FFF, it is of real interest to analyze, establish, and understand how these variables influence, especially in the case of fiber-reinforced polymeric composites about which there are no published analysis results. Investigations by many researchers have shown the effectiveness of the carbon fiber reinforcement (chopped) of thermoplastic polymers printed in FFF [1,2,6,7,8]. Liao et al. [6] reported the mechanical and thermal properties of chopped carbon fiber (CF)-reinforced polyamide 12 (PA12) composites fabricated by FFF with different carbon fiber mass fractions. The addition of 10 wt% carbon fibers into the PA12 matrix showed an increase in tensile strength (102.2%) and flexural strength (251.1%) than pure PA12. It was also found that the crystallization peak temperature and the degradation temperature increased by 3.46 °C and 7.50 °C, respectively, after adding 10 wt% carbon fibers [1]. In [9], the authors printed PLA composites reinforced with CF by varying the layers. The layers of carbon fiber PLA showed a maximum tensile strength of 1.16 times higher than that of pure PLA. Tian et al. [10] investigated the effect of FDM manufacturing parameters and carbon fiber content on the performance of samples produced from carbon-fiber reinforced composites. It has been found that composite PA has a better mechanical performance than ABS and PLA. Tekinalp et al. [11] compared the mechanical properties of parts made of carbon fiber-reinforced ABS fabricated by compression molding and FDM. The results indicated that both the strength and the modulus of the parts increased significantly with the increase in the carbon fiber content.

The effect of various manufacturing parameters, such as layer thickness, infill density, infill pattern, raster angle, and fiber orientation, on the mechanical properties was also investigated. Ning et al. [12] investigated the tensile characteristics of carbon fiber composites in terms of varying manufacturing parameters such as layer thickness, nozzle temperature, infill speed, and raster angle. Samples printed at a raster angle of—45°/45° showed a maximum modulus, whereas the maximum tensile strength was at an angle of 0°/90°. In [13], the authors explored the effect of manufacturing parameters such as infill density, layer thickness, and print speed on the mechanical properties of samples of Nylon material. It was found that infill density has the highest influence on the mechanical characteristics because flexural strength and UTS have maximum values at a 100% infill density. Naranjo-Lozada et al. [14] investigated the effects of infill density, infill patterns, and fiber volume fraction on the tensile properties of 3D-printed composites reinforced with continuous and chopped carbon fiber manufactured by fused filament fabrication (FFF). The results showed that infill density and infill patterns affect the mechanical behavior of the sample of chopped carbon fiber and the triangular pattern has a better tensile strength [15]. Gavali et al. [16] in their research found that an increase in the chopped carbon fiber in PLA composite led to an increase in flexural strength. After reinforcing with 10 wt% of carbon fiber, the strength increased to 67 MPa compared with 66 MPa of neat PLA. An increase in the percentage of carbon fiber of 15% led to a flexural strength of about 78 MPa [2]. In addition, the increase in the bending strength by adding different percentages of carbon fiber in the polyamide (PA) matrix was also found in the research carried out in the works [1,6,17,18]. Porter et al. [19] studied the effect of the infill orientation and density on the flexural properties of the PLA samples manufactured by FDM. The results showed that the percentage of 10 to 20% is the optimum infill for maximizing the stiffness-to-mass, but it is recommended to use a higher infill to avoid the drop off in flexural rigidity. Mishra et al. [20] researched the effect of different combinations of infill densities and infill patterns on the absorbed energy of the PLA 3D-printed samples. The results concluded that an 85% infill density for each infill pattern demonstrated the highest energy absorption. In [21], Tanveer et al. investigated the impact strength of the PLA samples with 50%, 70%, and 100% infill densities. The authors concluded that impact strength is direct proportional to infill density percentage. Aloyaydi et al. [22] investigated the influence of infill density on the microstructure and flexural behavior of PLA 3D printed using FDM. The result showed that the 80% infill density is the percentage that has the optimum strength and toughness. Terekhina et al. [23] showed that printed PA samples with a filling density above 60% have a significant increase in strength. The correlation between FFF parameters and the results was studied by Belei et al. [24]. It was found that the temperature of the printing bed and layer height are the most influential parameters that affected the mechanical performance of the printed samples. Maszybrocka et al. [25] stated that infill density is the main factor that influences the mechanical properties and found that strength properties increased with the increase in the filling density and they influenced both the core and outer layers filling density.

It is well known that the mechanical properties are influenced by the degree of crystallinity and, in the FFF, the temperature can directly affect the crystallinity of the printed part. Therefore, the thermal behavior of the composites reinforced with fibers are also investigated, although there are few studies. In the study of Liao et al. [6], it was demonstrated that the addition of carbon fiber in the PA12 composite reduced the thermal degradation of the matrix, and the authors concluded that carbon fiber acts as a thermal stabilizer, which protects the composite matrix. The thermal properties of the FDM polymeric Nylon 618, PLA, and PLA-reinforced carbon fiber in Kaur et al. [26] were studied. The 3D-printed carbon-based PLA exhibited better thermal behavior compared to the other polymeric materials. De Toro et al. [27] studied the thermal behavior and mechanical properties of the polyamide composite reinforced with 20% carbon fiber. The result showed the glass transition at 50 °C and the maximum degradation of the matrix at 450 °C. In [28], the addition of 20% carbon fiber to the ABS matrix led to a reduction in the glass transition temperature (from 110 °C to 105 °C) and also to a reduction in thermal stability by decreasing degradation on the set temperature (from 323 °C to 253 °C).

Carbon fiber PA composite, compared to ABS and PLA, has a wide field of use and superior mechanical performance, which justifies the need to research the application of PA reinforced with carbon fiber in FFF. Although the properties of various composite materials reinforced with various types of material have been studied, some fundamental properties of commercially available materials and uses in large-scale printing and in various fields have not yet been studied. The motivation of this paper resulted from the need to analyze the capacity of both FFF technology and commercially available fiber-reinforced composites to manufacture components (e.g., unmanned aerial vehicle components) subject to different types of stress [19,29]. Therefore, the purpose of this paper is the investigation of the infill density (ID) influence on the thermal and mechanical properties of commercially available PAHT CF15 composite, reinforced with carbon fiber (CF), used in the Fused Filament Fabrication (FFF) process. The influence of infill density (25%, 50%, 75%, 100%) on the mechanical performance of the samples was performed by a tensile test and three-point bending test. Microscopic analyses were performed to highlight the distribution and orientation of the carbon fibers in the polymer matrix, the defects in the microstructure of the filament, and those that occurred during the manufacturing process through FFF. The thermal behavior was characterized by Differential Scanning Calorimetry (DSC) and Thermogravimetric analysis (TGA) to obtain information about material transition, melting, and degradation.

## 2. Materials and Methods

All samples were printed with the BCN3D Epsilon W50 printer (BCN3D Technologies, Barcelona, Spain) with the parameters shown in Table 1. Preparation of the samples for manufacturing was carried out in the Cura—BCN3D software, 2.1.2 version (BCN3D Technologies, Barcelona, Spain). The 3D models of the samples for tensile and flexural tests were designed according to ASTM D638 [30] and ASTM D790 [31] standards, respectively. The dimensions of the Type I dogbone-shaped samples for tensile tests and flexural strength are presented in Table 2 and Table 3, respectively. All tensile tests were carried out at a 5 mm/min speed. The three-point flexural test was conducted using a distance between the two supporting rollers of 95 mm, with the supporting rollers diameter of 30 mm and the speed set to 10 mm/min.

The carbon fiber-reinforced polyamide composites, Ultrafuse PAHT 15, was used for printing the samples. The PAHT CF15 is a high-temperature polyamide-based filament filled with 15% chopped carbon fibers for Fused Filament Fabrication (FFF) [32]. PAHT CF15 is a high-performance 3D printing filament that allows the manufacture of parts that can be subjected to high thermal and mechanical loads [33]. The mechanical and physical properties of the Ultrafuse PAHT CF15 filament supplied by the manufacturer (BASF 3D Printing Solutions BV, Emmen, The Netherlands) for the flat XY-direction print in Table 4 are shown [33]. The filament was used in the printing process in the state delivered by the manufacturer, without being subjected to thermal procedures, being stored in its original packaging.

A total of 5 specimens were printed for each type of infill (25%, 50%, 75%, 100%) using a triangle infill pattern, resulting in 20 specimens for the tensile tests and 20 for the flexural ones, respectively. To evaluate the tensile properties of the samples, a WDW-150 S universal testing machine was used at a constant crosshead speed of 5 mm/min. For flexural properties in the three-point loading, the same testing machine was used. The crosshead speed and span length were 3 mm/min and 115 mm, respectively. The microstructure, the distribution and orientation of carbon fibers, and the distribution of voids both in the filament and the samples were investigated by optical microscopy. The samples and filament were cross-sectioned, embedded into resin, and polished using a 1 μm grit and 0.5 μm Al_2_O_3_ suspension. Using an Omnimet–Buehler microscope (Nikon, Tokyo, Japan), micrographs were taken on the sections perpendicular to the XY plane (cross-section) and the Z building direction (longitudinal section) with magnifications of 50× and 200×. Differential Scanning Calorimetry (DSC) was conducted under the nitrogen atmosphere using a NETZSCH DSC 200 F3 Maia (NETZSCH-Gerätebau GmbH, Selb, Germany) to highlight the glass transition, melting, and crystallization of the samples. DSC tests were carried out on the as-received filament and extruded material in accordance with ASTM D3418 [34] at a rate of 10 °C/min, cooling to −150 °C, and then heating to 500 °C. Thermogravimetric analysis (TGA) was performed on the NETZSCH TG STA 449F3 Jupiter (NETZSCH-Gerätebau GmbH, Selb, Germany) to identify the degradation temperature and weight loss. TGA was carried out from 20 °C to 500 °C at a heating rate of 10 °C/min according to the ASTM Standard E1131 [35]. TG analyses were performed in the temperature ranges of 20–100 °C, 100–300 °C, and 300–500 °C. NETZSCH Proteus Thermal Analysis software was used to process the data. It should be mentioned that the microstructural analyses were performed on the shell area of the samples, and for the DSC and TGA analyses, the mass of material was taken from the core area of the samples.

## 3. Results and Discussions

### 3.1. Mechanical Properties

#### 3.1.1. Tensile Strength of Carbon Fiber PAHT Composite

**Figure 1:**
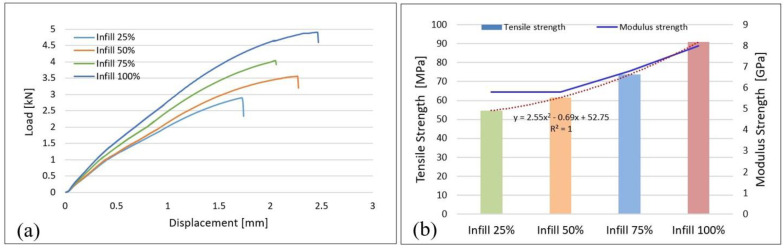
Results after tensile tests; (**a**) load–displacement curves; (**b**) tensile strength, modulus, and relationship between tensile strength and infill density.

In order to establish the influence of the infill density on the tensile strength of the samples manufactured by FFF, a comparative study was performed between the four types of infill. The data obtained during the tensile tests were processed, and the load–displacement curves as an average of the values for the five samples tested with infills of 25%, 50%, 75%, and 100% are shown in Figure 1a.

For each infill density, a linear behavior can be ascertained between the load and displacement up to the point of yield strength (plastic deformation of the material begins) and up to the point where the load is maximum and failure and breaking occur. From Figure 1a, it can be seen that the increase in the infill density determines the increase in the load and influences the displacement. For the samples with a 100% infill, the highest load (4.9 kN) and displacement (2.54 mm) are registered, and those with a 25% infill have the lowest values (2.9 kN and 1.74 mm, respectively), which confirms the influence of the infill density on tensile strength, results that are consistent with those published in [13,14]. This is justified by the fact as the infill density increases, the content of polymeric material increases (which determines the air gaps in the material to decrease) and implicitly the cross-sectional area. Therefore, the parts manufactured by FFF can support a higher load as the infill percentage increases.

Samples with a 75% infill have a smaller maximum displacement (2.22 mm) than those with 50% (2.28 mm) even if the maximum sustained load (4.03 kN) is higher than that at a 50% infill (3.56 kN). It has been demonstrated [21] that the outer layers (shell), which are denser, provide a resistance to crack propagation. Under action load, during failure of the sample, the cracks propagate from shell to core (inner layers) [21,36]. The presence of manufacturing defects both within the shell and core, such as voids and gaps, as shown in micrographs of the samples with 75% infill density, leads to the rapid propagation of cracks and the decrease in interlayer cohesion, as reported in [24,36], which determine breaking of the samples with a 75% infill after a lower displacement than 50% ID.

In order to determine the tensile strength, an average of the values of the maximum stresses and of the modulus of elasticity of the five samples from each type of infill (25%, 50%, 75%, 100%) was calculated. The plot in Figure 1b shows that as the infill density increases, the tensile strength also increases. These results are in line with those published in [2,13,14,19,21]. Therefore, samples with a 25% infill have a lower strength, of 56.4 MPa, and an elasticity modulus of 5.8 GPa, and those with a 100% infill have a strength that increases to 90.8 MPa and a modulus that increases to 8 GPa. The relationship between the tensile strength and the degree of filling is indicated by an order 2 polynomial, as shown in Figure 1b.

Figure 1b shows that for samples with 25% and 50% infills, the differences between the values of maximum tensile strength are small (54.6 MPa and 61.6 MPa, respectively, a difference of 7 MPa) and the tensile modulus is the same, 5.8 GPa. Taking these results into account, it can be stated that the 25% infill can be used instead of the 50% infill, if the operating conditions of the components allow, because the manufacturing cost is lower using less material and a shorter print time.

#### 3.1.2. Flexural Strength of the Carbon Fiber PAHT Composite

**Figure 2:**
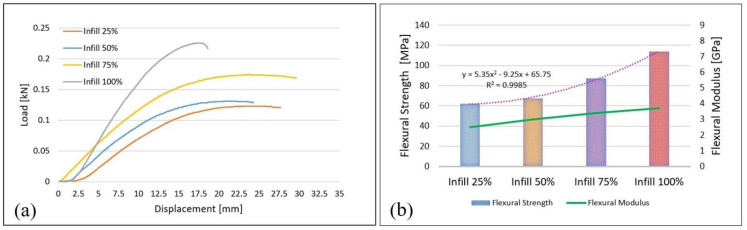
Results after three−points flexural tests: (**a**) flexural test load–displacement curves; (**b**) flexural strength, modulus, and relationship between flexural strength and infill density.

The flexural behavior of the samples with the four types of infill percentages in Figure 2a is shown. Load–displacement curves show that samples with a 100% infill have the highest strength-to-maximum load of 0.23 kN. In contrast, compared to other samples, they have the smallest displacement, 18.6 mm. The analysis of the curves shows that the samples with 25% and 50% infills (Figure 2a) withstand very close loads, with the differences between the values being very small, that is, 0.13 kN for 50% and 0.12 kN for 25% infill densities.

It is known that in flexural tests, the exterior surfaces are most subject to stress. Therefore, the flexural strength will be determined by the strength of the intact areas/layers. In the case of tensile tests, all layers (material) are subjected to the same stress and, therefore, breakage/failure will be initiated when the layer/material on the layer reaches the tensile stress limit. The higher displacement of the samples with an infill of 25% (maximum displacement of 25.8 mm) under the action of lower loads could be influenced by the existence in the microstructure at the top, bottom, and lateral surfaces of the samples of a greater number of voids and the occurrence of microcracks as well.

The average values of the flexural strength as well as the values of the modulus corresponding to the four infill densities are shown in Figure 2b. In Figure 2b, it can be seen that the samples with a 100% infill have the highest flexural strength (114 MPa) and the highest flexural modulus (3.7 GPa). Between samples with a 25% infill density and those with 100%, it is found that there is an increase in bending strength of 83.3%, which means that infill density has a major contribution, as found in previous studies [2,6,13,19,21]. In contrast, 50% infill density samples have a strength of only 8.7% higher than those with a 25% infill. In order to express the relationship between flexural strength and infill density, the polynomial equation of order 2 was chosen (Figure 2b) as it describes with the greatest precision the relation between them (R2 is 0.9985, in contrast with 0.929 in the case of a linear relation) [25,37]. As in the case of tensile tests, it is found that there are small differences between the values of flexural strength for samples with infill densities of 25% and 50%, respectively. Therefore, to reduce manufacturing/printing time and material and energy consumption, an infill with a density of 25% can be used instead of 50%.

#### 3.1.3. Microstructural Characteristics of the Carbon Fiber PAHT Composite

**Figure 3:**
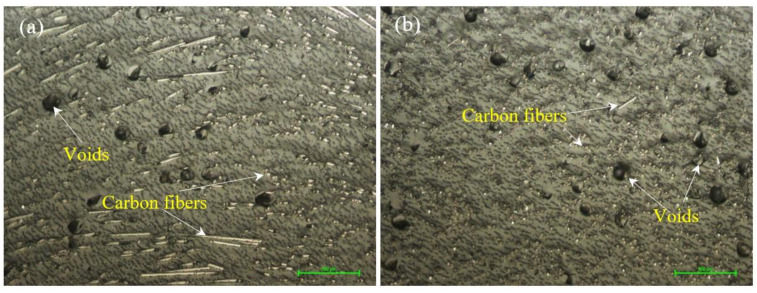
Micrographs of the PAHT CF15 filament. (**a**) Longitudinal section/view (200×); (**b**) cross-section (200×).

Microstructural analyses, both for the filament and for printed samples, were performed to highlight aspects specific to 3D printing, namely the distribution and orientation of fibers in the polymer matrix, the presence of voids and deposition defects, the appearance of microcracks, and the influence of the infill percentage on the microstructure. Figure 3 shows the longitudinal (Figure 3a) and cross-sectional (Figure 3b) micrographs of the PAHT CF15 filament. Figure 3 shows that the distribution of carbon fibers in the polymer matrix is approximately uniform. Regarding the orientation of the carbon fibers, it is found that they are not entirely along the filament but rather have a random orientation, as can be seen in Figure 3a. In both sections, it is found that the filament contains, in addition to carbon fiber, voids (manufacturing defects of the filament). The voids, in turn, can influence the quality of the microstructure of the deposited material and subsequently the mechanical properties of the samples.

**Figure 4:**
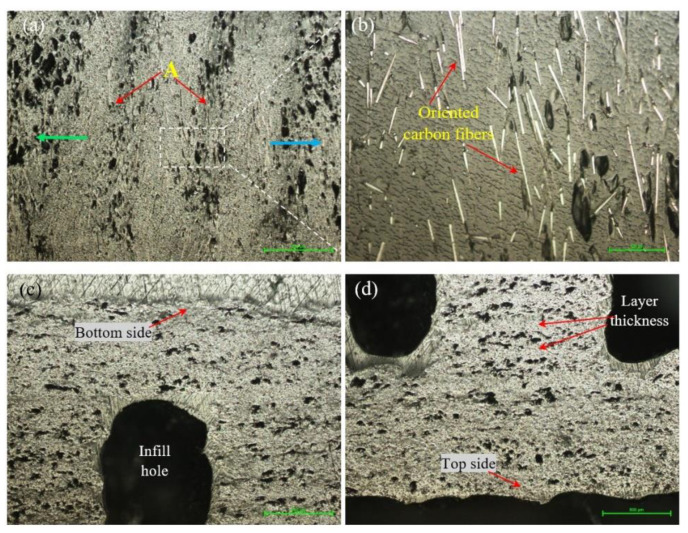
Micrographs of the samples with 25% infill density. (**a**,**b**) Longitudinal section (200×); (**c**) cross-section top side and (**d**) bottom side (50×).

Figure 4 shows the microstructure of the samples manufactured by FFF with an infill density of 25%. Figure 4a shows the microstructure, in the longitudinal section, on the exterior (lateral) wall of the sample. It can be seen that, after printing, the porosity increases toward the outside of the specimen (blue arrow) but also toward the inside where the hole related to the infill is found (green arrow). It is also found that at the boundary between the successive beads (bead–bead interface), there are linear areas with a higher porosity (voids) than those of the deposited layer (Figure 4a, areas marked with A). This phenomenon was also noticed in the published results of previous studies [1,6,7]. An increase in porosity is also seen in the printing direction (Z direction) from bottom (Figure 4c) to top (Figure 4d) [38]. A factor that could lead to an increase in the porosity (voids) is the decrease in the pressure/weight of the layers on those already deposited as the deposition of the extreme layers approaches, and, consequently, on the extreme layers (top and lateral layers), only the atmospheric pressure from the printing chamber acts. In addition, the temperature distribution in the deposited layers is another influencing factor, as mentioned in the studies presented in [39,40].

Figure 4b shows a detail from Figure 4a where it can be seen that, after melting the filament and passing the material through the nozzle, a reorientation of the carbon fibers takes place, most of them being oriented along the deposition direction (along the deposited beads), as also presented in the papers [1,18,41]. The direction of deposition, both for the samples tested at tensile and the flexural strengths, was along the samples (in the XY plane).

**Figure 5:**
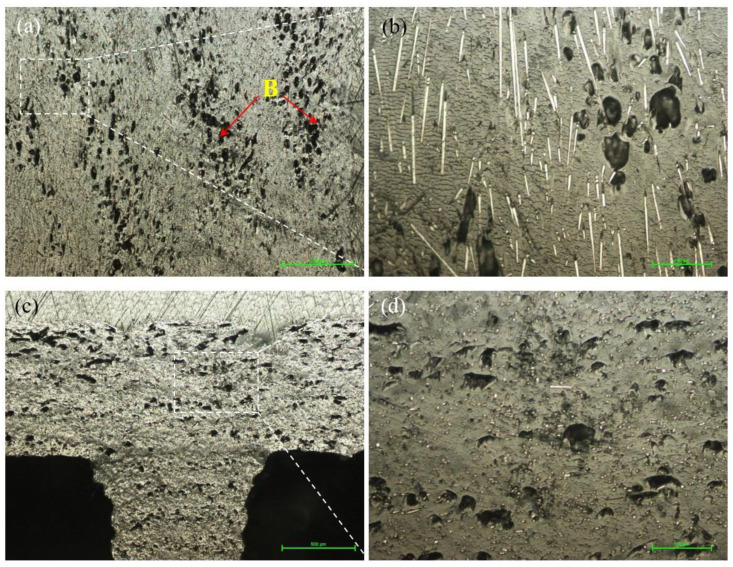
Micrographs of the samples with 50% infill density. (**a**,**b**) Longitudinal section (200×); (**c**,**d**) top side of the cross-section at 50× and 200×.

**Figure 6:**
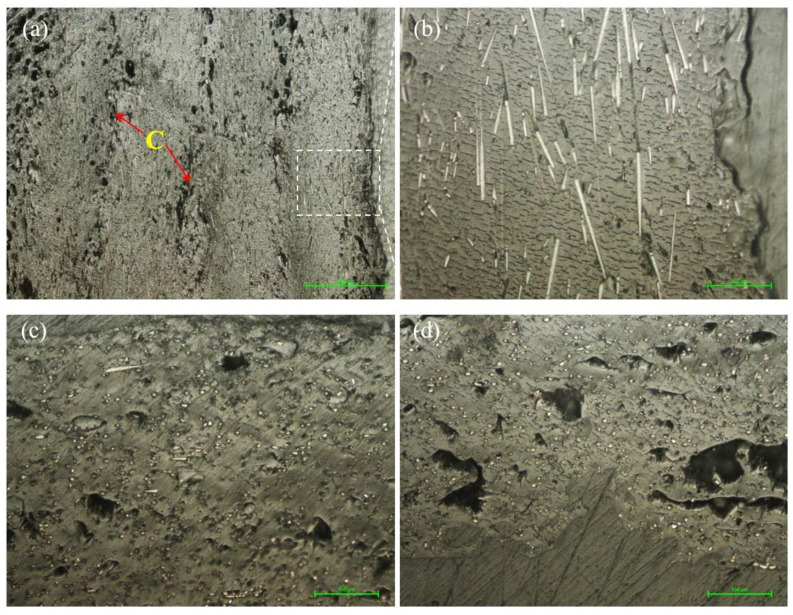
Micrographs of the samples with 75% infill density. (**a**,**b**) Longitudinal section (200×); (**c**,**d**) bottom and top side of the cross-section (200×).

The microstructure of the samples manufactured with an infill density of 50% is presented in Figure 5. In the longitudinal section made in the lateral part of the sample (Figure 5a, areas marked with B), it is found that the area of porosity/defects (voids) between the successive beads in the XY plane is almost the same as for 25% samples but is much more pronounced than for 75% infill density ones (Figure 6a). As in the case of samples manufactured with a 25% infill, on the cross-section (Figure 5c,d), there is a higher porosity in the upper part of the samples.

In Figure 5b, carbon fibers are mostly oriented in the direction of longitudinal deposition, while in the cross-section (Figure 5d), it can be seen that there are extremely few carbon fibers, which have a different orientation. As previously mentioned, the presence of defects (voids, pores) at the level of the microstructure influences the mechanical properties. Consequently, the mechanical performances of samples with a 50% infill close to those with an infill density of 25% is justified.

Compared with the microstructure of the samples manufactured with 25% and 50% infill densities, respectively, the 75% infill density samples show a microstructure with fewer voids at the boundary between the successive material deposition (bead–bead interface) (Figure 6a), which means that there is a better adhesion between them. In addition, the reduction in defects can be seen in the micrographs of the cross-section (Figure 6c,d), but there remains a more pronounced porosity in the top part (Figure 6c) of the samples compared to the bottom one (Figure 6c). As in the case of the 25% and 75% infill density samples, the reorientation of the carbon fibers along the direction of the deposition beads is found (Figure 6b), after the filament melts and the molten material passes through the extruder.

**Figure 7:**
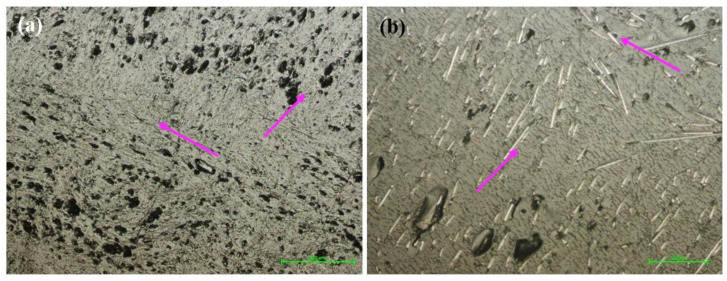

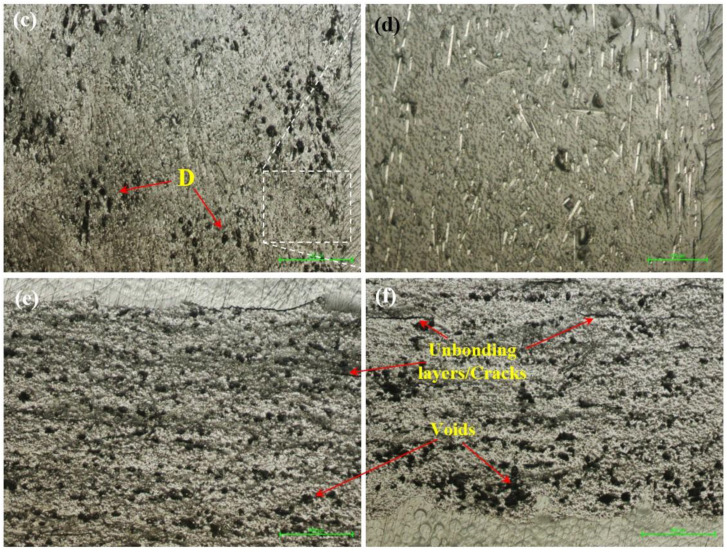
Micrographs of the samples with 100% infill density. (**a**,**b**) Longitudinal section from the interior of the sample (50×, 200×); (**c**,**d**) longitudinal section from the lateral side of the sample (50×, 200×); (**e**,**f**) bottom and top side of the cross-section (50×).

Figure 7a,b show the microstructure inside (core) the samples manufactured by FFF with an infill density parameter of 100%. The different orientations of the carbon fibers are found (Figure 7a,b, purple arrow), depending on the deposition direction of the molten material bead. The microstructure on the lateral side of the sample (Figure 7c,d), compared to those of the samples with 25%, 50% and 75% infills, contains fewer voids between beads (Figure 7c, D areas) and the carbon fibers are oriented (Figure 7d) along the direction of the printed beads. In the cross-section from Figure 7e,f, it is found that the voids in the microstructure have a uniform distribution, that is, they respect to a certain extent a pattern and they are on the same line. Both in the bottom side of the section (Figure 7e) and in the top one (Figure 7f), interlayer cracks (interlaminar interface) were identified and the porosity increased from the bottom to the top side of the sample.

A common printing defect in FFF of fiber-reinforced composites is the formation of voids [1,6,7]. However, the defects of the voids type contained in the filament are transformed into voids in the printed beads. Previous studies [1,6,7] have demonstrated that voids (triangular or different in shape) formed during FFF manufacturing will act as stress concentrators that will reduce the mechanical performance of the parts. Therefore, a thorough understanding of the structure and mechanics of FFF of carbon fiber composites is required. Based on the microstructure results for the four infill densities used in the manufacture of PAHT CF15 specimens by FFF technology, it can be mentioned that an increase in infill density leads to a decrease in the voids at the bead–bead interface. However, in specimens with a 100% infill density, the increase in interlayer defects (unbonding of layers or the appearance of cracks) is found. In the future, the authors intend to continue research in order to reduce the formation of interbeads and interlayer voids by decreasing the thickness of the deposited layer because a thinner layer could increase the adhesion between beads and layers, as well as mechanical properties, as mentioned in the studies presented in [7,12].

#### 3.1.4. Thermal Behavior of Carbon Fiber PAHT Composite

**Figure 8:**
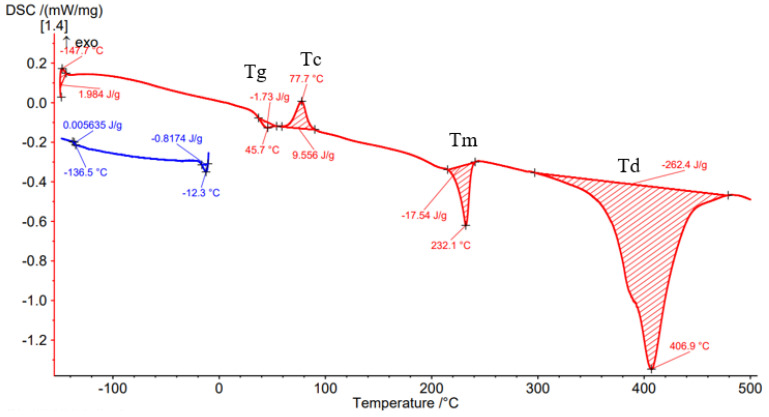
DSC thermogram for PAHT CF15 filament.

**Figure 9:**
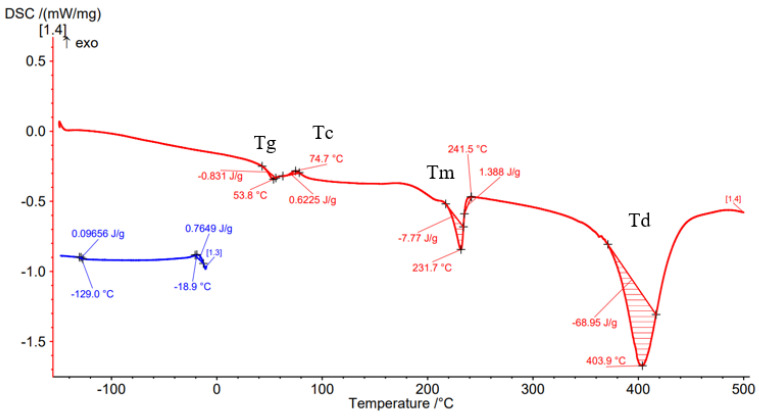
Results of DSC analysis for sample with 25% infill density.

**Figure 10:**
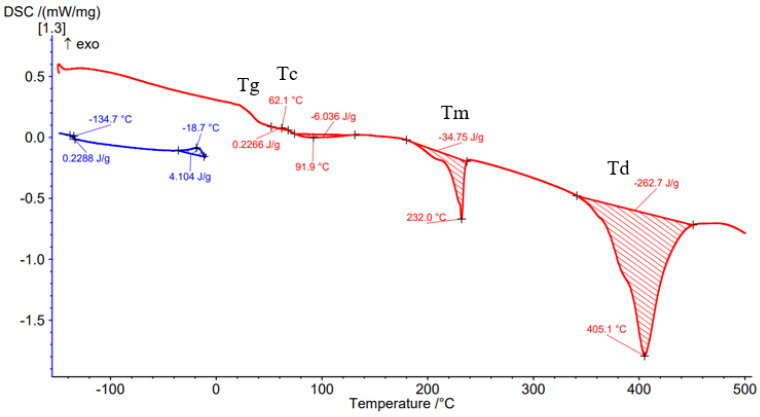
DSC analysis for sample with 50% infill density.

**Figure 11:**
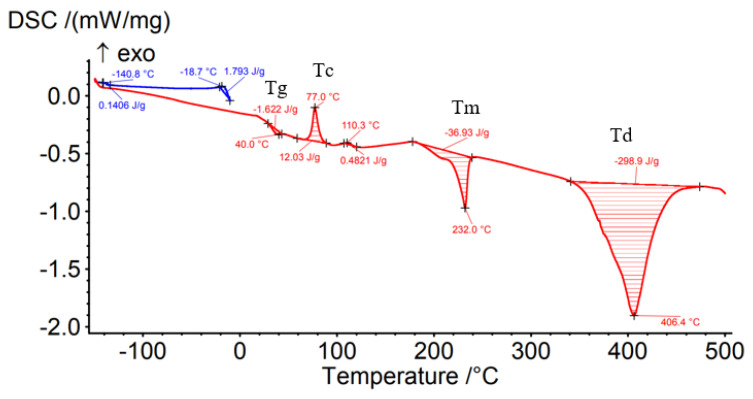
DSC thermogram for sample with 75% infill density.

**Figure 12:**
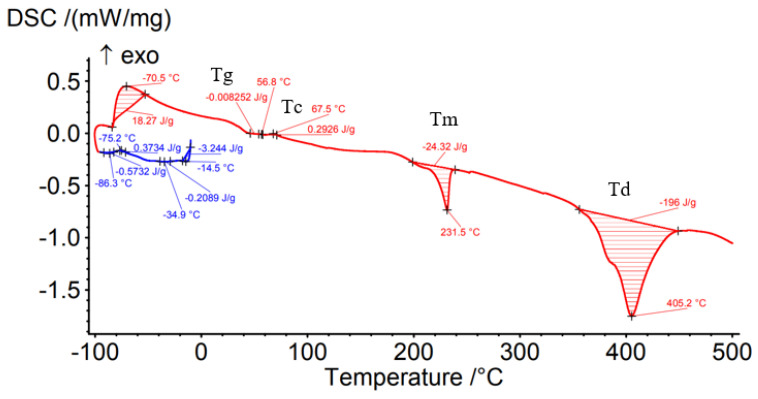
DSC curves related to sample with 100% infill density.

Differential Scanning Calorimetry (DSC) was carried out to determine the effect of the processing conditions on the filament material as well as on the material deposited by the FFF process. Figure 8, Figure 9, Figure 10, Figure 11 and Figure 12 show the DSC curves obtained from the analyses both of the PAHT CF15 filament (Figure 8) and for the samples manufactured by FFF with the four infill densities (25%, 50%, 75%, and 100%, respectively) (Figure 9, Figure 10, Figure 11 and Figure 12). A comparison of the DSC curves highlighted the differences between their thermal characteristics. On the heating curves (red curves), all samples exhibited significant endothermic peaks due to melting (Tm). Melting temperature (Tm) is an important indicator in setting the printing temperature, and the values obtained (Table 5 and Table 6) are close to those mentioned by the filament manufacturer (Table 4), the maximum difference being 2.5 °C in the case of samples with an infill density of 100%. In addition, melting peaks are characteristic of semi-crystalline materials and indicate that the structure of the samples is crystalline (melting of the crystalline structure).

**Figure 13:**
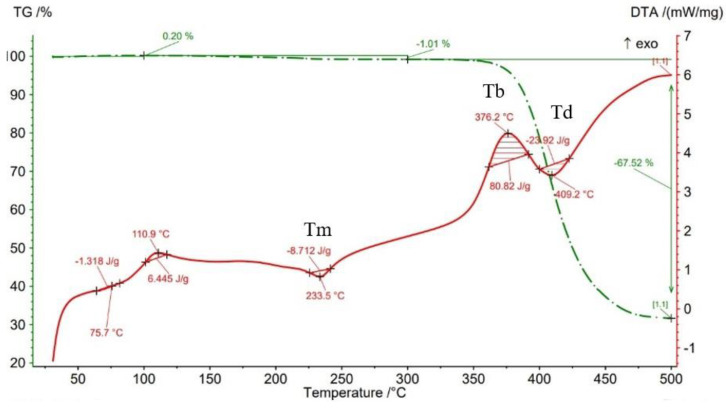
TGA curves for PAHT CF15 filament.

**Figure 14:**
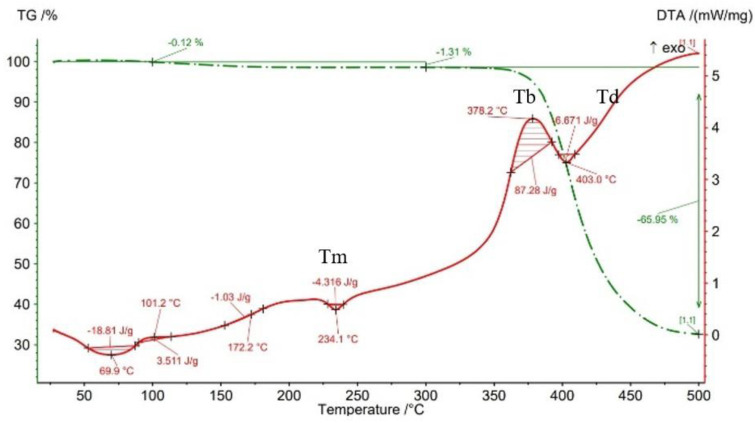
TGA results for sample with 25% infill density.

**Figure 15:**
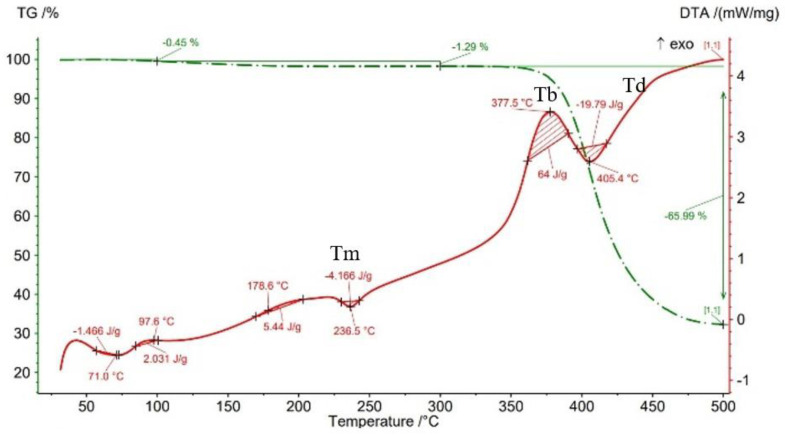
Thermogravimetric analysis curves for sample with 50% infill density.

**Figure 16:**
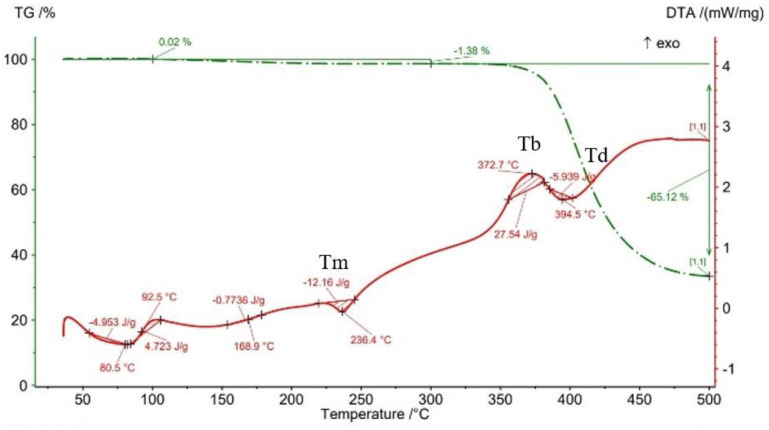
TGA results for sample with 75% infill density.

**Figure 17:**
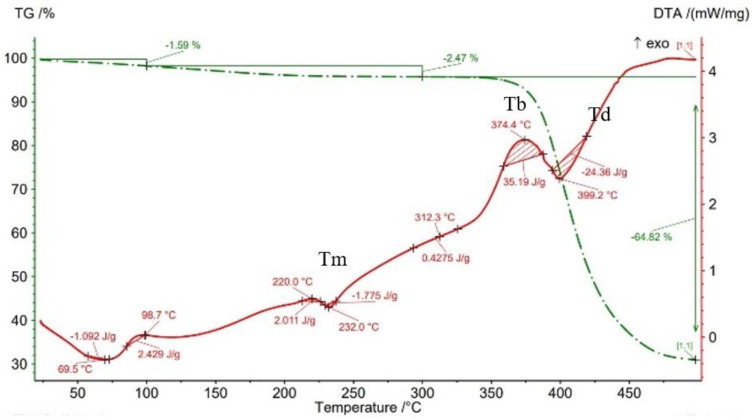
Thermogravimetric analysis results for sample with 100% infill density.

The glass transition temperature (Tg) is the temperature at which the material turns from a glassy, rigid material to a soft, rubbery material due to the molecular motion of amorphous chains [42]. From the DSC curves (Figure 8, Figure 9, Figure 10, Figure 11 and Figure 12) and the data presented in Table 5, it is found that the temperature Tg decreases as the infill density increases; the lowest glass transition temperature is recorded for the specimens with an infill density of 100% (Figure 12, Table 5). It is well known that carbon fiber has a higher thermal conductivity, which influences the thermal stability of the matrix. The increase in the thermal mobility of the polymer chain due to the chopped carbon fibers leads to the decrease in the glass transition temperature; thus, the transformation from the glassy phase to the rubbery one takes place at a lower temperature. The decrease in glass transition temperature (Tg) as the carbon fiber content increases was also mentioned in [7,28,43]. Therefore, the increase in infill density also involves, in addition to a larger volume of polymeric matrix, the increase in the amount of carbon fiber that causes the decrease in Tg as the infill density increases. It can be stated that infill density, as a 3D printing parameter, influences the thermal properties of the material. Exothermic peaks after the glass transition temperature (Tg) represent the cold crystallization temperature (Tc) (Figure 8, Figure 9, Figure 11 and Figure 12). The cold crystallization is an incomplete crystallization because of the fact that during the fast cooling from the molten state, the filament and the printed samples do not have enough time to crystallize. Upon reheating above Tg, the thermal energy provides sufficient molecular mobility to allow the polyamide to crystallize. For samples with a 50% infill density (Figure 10), the cold crystallization peak is very small; therefore, it has the lowest imperfect crystallization. Small peaks identified before the melting temperature for printed samples (Figure 9, Figure 10, Figure 11 and Figure 12) refer to the recrystallization melting temperature. This temperature can be caused by morphological changes such as the completion of crystallization and the refinement of crystals, as stated in the paper [42] too. From the curves in Figure 8, Figure 9, Figure 10, Figure 11 and Figure 12, it is found that exceeding the melting temperature (Tm) has the effect of starting the degradation of the material, and the endothermic peaks highlight the complete degradation of the material (degradation temperature, Td). Table 5 shows the values of the most important peaks (Tg, Tc, Tm, Td) on the DSC heating curves in Figure 12, Figure 13, Figure 14, Figure 15, Figure 16 and Figure 17.

The results of thermogravimetry analyses (TGA) are shown in Figure 13, Figure 14, Figure 15, Figure 16 and Figure 17 and Table 6. Figure 13 displays the TGA curve of the PAHT CF 15 filament and the results show a weight gain of 0.20% up to the temperature of 100 °C. It is known that polyamide (PA) [32,44] tends to absorb water readily and, therefore, weight gain (Figure 13) is a consequence of water absorption. The water content influences the glass transition in the sense that as the water content increases, the glass transition will be at a lower temperature, as also mentioned in the paper [44]. This means that the filament should be dried before it can be used in 3D printing. Studying the influence of moisture on the glass transition temperature (Tg) of the material tested is an objective of future papers. In the temperature range of 100–300 °C, the mass loss is 1.01% because of water evaporation, melting (at 233.5 °C, endothermic peak, Tm), and also the beginning of the matrix decomposition (after exceeding the melting temperature). The analysis of the PAHT CF15 filament’s thermogram (Figure 13) shows that another significant mass loss effect begins at over 350 °C, in the temperature range of 300–500 °C. Above this temperature, the process of accentuated damage of the matrix begins with burning of the material (at 376.2 °C, exothermic peak, Tb) until the complete destruction (at 409.2 °C, endothermic peak, Td), where the mass loss is 67.52% (Figure 16 and Figure 17, Table 6). It can be stated that the limit of thermal stability in given conditions is about 346 °C (more exactly, 345.75 °C).

Regarding the TG analyses related to the samples manufactured by FFF, it is found that up to 100 °C, all specimens have low mass loss due to water evaporation, except for the one with a 75% infill density, which registers an extremely small increase of 0.02%. The 0.02% increase in mass may be due to the absorption of water, which also leads to a decrease in the glass transition temperature. For the 100% infill density sample, the lowest glass transition temperature, of 32.9 °C, is found (Figure 12, Table 5). The specimens with an infill density of 100% have the highest mass loss, of 1.59% (Figure 17), and those with the lowest loss are those with an infill of 25% (0.12%). It can be concluded that at a 100% infill, which involves a greater volume of polyamide (matrix) and a greater volume of carbon fiber, the mass losses will be higher as well. In the temperature range of 100–300 °C, there are mass losses of over 1% for all four infill densities, and the samples with an infill of 100% have the highest loss, 2.47% (Figure 17). However, the lowest mass loss is related to the 50% infill density sample (1.29%) (Figure 15, Table 6). The thermograms in Figure 14, Figure 15, Figure 16 and Figure 17 show that in the temperature range of 300–500 °C, there are higher mass losses, more precisely above the temperature of about 345 °C (344.75 °C). As in the case of the TG analysis of the PAHT CF15 filament (Figure 13), exceeding the temperature of 345 °C determines the beginning of the accentuated degradation of the material for all the four infill densities. The highest burning temperature is recorded for the 25% infill density sample (378.2 °C) (Figure 14, Table 6) and the lowest for the 75% infill density one (372.7 °C) (Figure 16). The complete destruction of the matrix is at a maximum temperature of 405.4 °C recorded for the sample with a 50% infill density (Figure 15), which also has the highest mass loss, of 65.99%, and the lowest temperature of complete degradation is related to the specimen with a 75% infill density (394.5 °C). Compared to the 25% and 50% infill density (ID) samples, the 75% and 100% infill density ones have the lower melting temperatures, burning, complete degradation, and also lower mass losses (Table 6, Figure 14, Figure 15, Figure 16 and Figure 17). One explanation could be that in the samples with IDs of 75% and 100%, at the microstructure level, from the areas the material was taken for analysis (DSC, TGA), the volume of the voids and/or the volume of the carbon fiber distributed in the polymer matrix are higher than those at 25 and 50%, respectively, which would mean a smaller volume of polyamide, which, in turn, requires a lower amount of energy (temperature) for both melting and decomposition and, therefore, after degradation, a lower mass loss is obtained.

## 4. Conclusions

This paper presents a study on the thermal and mechanical behavior of fabricated samples of polyamide reinforced with 15% carbon fiber, PAHT CF15, commercially available, using the additive manufacturing process Fused Filament Fabrication (FFF). The samples were manufactured with 25%, 50%, 75%, and 100% infill densities (IDs) and subjected to tensile and three-point flexural tests, DSC and TGA thermal analyses, as well as microstructural analyses.

- The results of three-point flexural and tensile tests showed that the infill density percentage has a major influence on mechanical properties. As the infill density increases, so does the tensile and flexural strength.
- It was found that for the samples with 25% and 50% infill densities, the differences between the maximum tensile and flexural strengths are small, 7 MPa and 5.4 MPa, respectively. It is well known that reducing infill densities results in lower printing costs and times. Therefore, if the conditions of use of the components made of PAHT CF15 allow, using a 25% infill density in the FFF process could be seen as an option.
- The microstructure analysis showed that after the printing process, the carbon fibers in all the PAHT CF15 samples are preferentially aligned along the printing direction, and it does not eliminate the voids present in the filament material. It was also found that the content of voids between successive deposition beads is higher the lower the infill density. The presence of a high volume of voids (defects) in the structure of the material leads to a decrease in the mechanical properties of the components manufactured by FFF. Therefore, it is necessary to reduce or eliminate voids by adopting in situ post-processing techniques or changing the printing parameters.
- DSC analyses indicated that both the PACH CF15 filament and the printed samples show glass transition, cold crystallization, and melting and degradation peaks. It was found that the infill density percentage influences the glass transition temperature (Tg); therefore, the increase in ID determines the decrease in the transition temperature from the glassy to the rubbery phase (Figure 8, Figure 9, Figure 10, Figure 11 and Figure 12, Table 5). The highest value of Tg, 49.6 °C, was recorded for samples with an ID of 25%, while for those with an ID of 100%, Tg is 32.9 °C.
- The results of the TGA analysis of the PAHT CF5 filament showed that exceeding the temperature of 345.75 °C (thermal stability limit) determines the beginning of the accentuated degradation process of the material with significant mass loss (Table 6, Figure 13, Figure 14, Figure 15, Figure 16 and Figure 17). In the case of samples manufactured with the four infill densities, the thermal stability limit is close to the filament, namely at 344.75 °C (in the case of specimens with IDs of 75% and 100%). It was found that the increase in the percentage of ID causes a decrease in the thermal degradation temperature (onset temperature). Samples with IDs of 75% and 100% recorded a temperature of 344.75 °C and those with IDs of 25 and 50%, 348.5 °C and 349.6 °C, respectively (Figure 13, Figure 14, Figure 15, Figure 16 and Figure 17).

## Figures and Tables

**Table 1:** Manufacturing parameters for the PAHT CF15 composite samples.

Parameter	Value	Unit
Filament diameter	2.85	[mm]
Layer height	0.2	[mm]
Infill density	25; 50; 75; 100	[%]
Print speed	50	[mm/s]
Extrusion temperature	260	[°C]
Building plate temperature	90	[°C]
Nozzle diameter	0.6	[mm]
Number of lower and upper layers	8	
Number of shell contours	4	
Infill pattern	Triangle (0°/60° with respect to load direction)	

**Table 2:** Dogbone-shaped samples dimensions subjected to tensile tests.

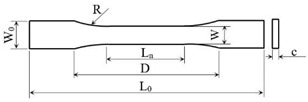	**Length** **Overall** **L_0_ [mm]**	**Distance** **between Grips** **D [mm]**	**Length of Narrow** **Section** **L_n_ [mm]**	**Radius of Fillet** **R [mm]**	**Width** **W_0_ [mm]**	**Width of Narrow Section** **W [mm]**	**Thickness** **c [mm]**
	165	115	57	76	19	13	4

**Table 3:** Samples dimensions subjected to flexural tests.

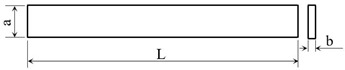	**Length** **L [mm]**	**Width** **a [mm]**	**Thickness** **b [mm]**
	200	20	4

**Table 4:** Mechanical and physical properties of the Ultrafuse PAHT CF15 filament [33].

Mechanical and Physical Properties	Unit	Ultrafuse PAHT CF15		Testing Method
		Dry Specimen	Conditioned Specimen	
Tensile strength	[MPa]	103.2	62.9	ISO 527
Elongation at Break	[%]	1.8	2.9	ISO 527
Young’s Modulus	[MPa]	8386	5052	ISO 527
Flexural Strength	[MPa]	160.7	125.1	ISO 178
Flexural Modulus	[MPa]	8258	6063	ISO 178
Flexural Strain at Break	[%]	2.4	No break	ISO 178
Melting temperature	[°C]	234		ISO 11357-3
Glass Transition	[°C]	70		ISO 11357-2

**Table 5:** Differential Scanning Calorimetry (DSC) results.

Samples	Glass Transition Temperature Tg [°C]	Crystallization Temperature Tc [°C]	Melting Temperature Tm [°C]	Full Degradation Temperature Td [°C]
Filament PAHT CF15	38.5	77.7	232.1	406.9
Sample 25% ID	49.6	74.7	231.7	403.9
Sample 50% ID	34.6	62.1	232.0	405.1
Sample 75% ID	33.6	77.0	232.0	406.4
Sample 100% ID	32.9	67.5	231.5	405.2

**Table 6:** Thermogravimetric analysis results for PAHT CF15 filament and printed samples.

Samples	Softening Temperature Ts	Melting Temperature Tm	Burning Temperature Tb	Full Degradation Temperature Td	Mass Change 20–100 °C	Mass Change 100–300 °C	Mass Change 300–500 °C
	[°C]	[°C]	[°C]	[°C]	[%]	[%]	[%]
Filament PAHT CF15	…	233.5	376.2	409.2	+0.20	−1.01	−67.52
Sample 25% ID	172.2	234.1	378.2	403.0	−0.12	−1.31	−65.95
Sample 50% ID	178.6	236.5	377.5	405.4	−0.45	−1.29	−65.99
Sample 75% ID	168.9	236.4	372.7	394.5	+0.02	−1.38	−65.12
Sample 100% ID	220.0	232.0	374.4	399.2	−1.59	−2.47	−64.82

## Data Availability

Not applicable.
